# Management of Type 3 Acromioclavicular Joint Dislocation: Comparison of Long-Term Functional Results of Two Operative Methods

**DOI:** 10.5402/2012/580504

**Published:** 2012-06-13

**Authors:** Hari Kovilazhikathu Sugathan, Ronald Martin Dodenhoff

**Affiliations:** ^1^Department of Orthopaedics, South Tyneside Hospital, South Shields NE34 0PL, UK; ^2^Department of Orthopaedics, Princess Royal Hospital, Telford TF1 6TF, UK

## Abstract

*Introduction*. Treatment of Rockwood Type 3 Acromioclavicular joint dislocation is controversial. We compared the long-term functional outcome of early repair of coracoclavicular ligament and internal fixation (Tension Band Wiring) with delayed reconstruction by modified Weaver-Dunn procedure for Type 3 dislocations. *Method*. Retrospective analysis of case records and telephone review to assess the long-term functional outcome by patient satisfaction and Oxford shoulder score. *Results*. We had 18 cases of Type 3 Acromioclavicular dislocations over a period of 10 years. 7 cases had Tension Band Wiring and 11 cases had modified Weaver-Dunn procedure. Early repair group has higher risk (71%) of post operative complications compared to that of the delayed reconstruction group (9%). All 5 patients who developed postoperative complications in the early repair group required a second operation for metal work removal. Long-term functional results of both groups were comparable in terms of Oxford shoulder score and patient satisfaction. *Conclusions*. We recommend modified Weaver-Dunn procedure for failed conservative management of Grade 3 Acromioclavicular joint dislocation for the following reasons (1). better short-term functional outcome, low risk of complications and hence faster recovery (2). no need for a second surgery.

## 1. Introduction

The effectiveness of surgery for complete Acromioclavicular Joint (ACJ) dislocation is controversial. Availability of multiple techniques and variable results in the literature makes the treatment choice difficult. Rockwood identified six types of injuries [1]. Types 1 and 2 are incomplete injuries and are treated nonoperatively. Types 3 to 6 are complete injuries. Majority of the orthopaedic surgeons will agree for surgical treatment of types 4–6 ACJ dislocation [2]. As for type 3 AC dislocation both early surgical treatment and nonsurgical treatment initially with late reconstruction if necessary have gained support. But a satisfactory surgical technique has not been developed yet [3, 4]. Acromioclavicular fixation in acute complete ACJ dislocations has given excellent results in literature [5, 6]. Calvo et al. found that the clinical results of type III injuries managed operatively or nonoperatively were comparable [7].

**Figure 1:**
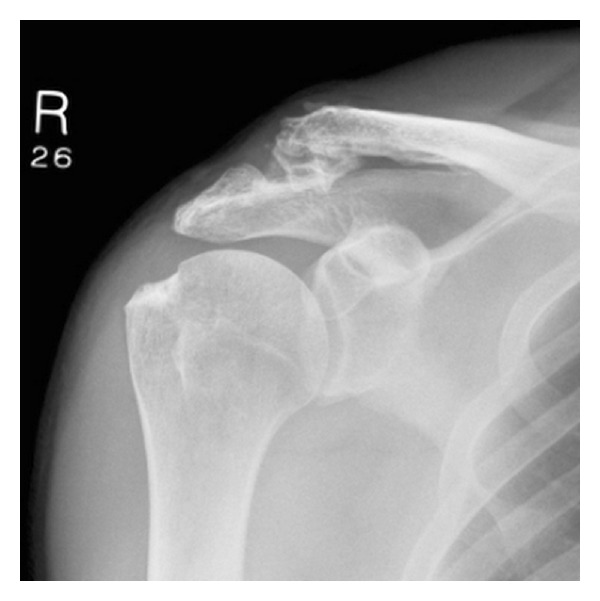
Chronic Type 3 ACJ dislocation.

When patients are seen more than 6 weeks after the initial injury, ACJ dislocation is considered to be chronic (Figure 1) because there is either partial or total resorption of the coracoclavicular (CC) ligaments. This makes direct ligament repair insufficient to stabilize the ACJ, and most authors recommend augmenting the repair [8]. The most popular and widely used CC ligament reconstruction technique for chronic injuries was originally described by Weaver and Dunn (WD) in 1972 [9].

 Open Reduction and Internal Fixation (ORIF) with Tension Band Wiring (TBW) (early repair for acute injuries) and modified Weaver-Dunn procedure (delayed reconstruction for chronic injuries) are the two procedures analysed in this study. We have compared the long-term functional outcome of early repair (TBW) with delayed reconstruction (modified WD procedure) for Type 3 ACJ dislocations.

## 2. Materials and Method

 Retrospective review of case notes of 18 patients with Type 3 ACJ dislocation, admitted for stabilization procedure over a period of 10 years at Telford Hospital, was done. 11 cases had modified WD procedure and 7 patients had ORIF with TBW. Telephone review of all the 18 cases was conducted and long-term functional outcome was assessed with Oxford shoulder Score [10]. Patient satisfaction was also recorded at the time of telephone review in terms of strength of the shoulder, appearance of shoulder and whether the patient was able to return to the preinjury level of activity or not.

## 3. Surgical Techniques

**Figure 2:**
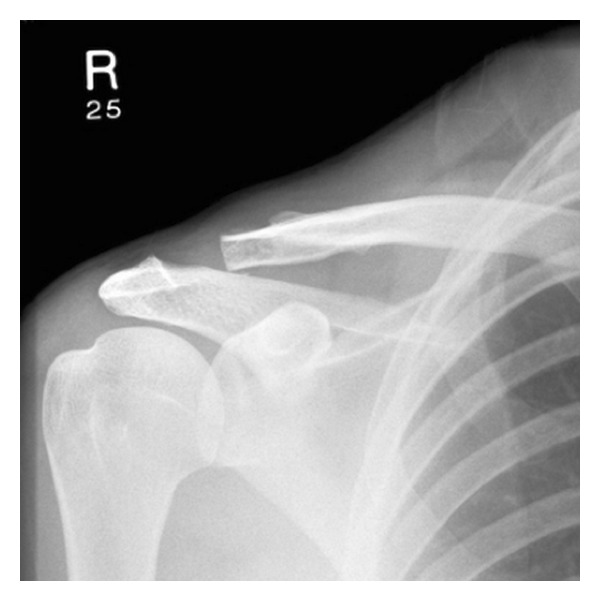
Two weeks post op modified WD procedure.

Modified Weaver-Dunn technique (described by Copeland in 1995) was used in the first group [11] (Figure 2). A 5 cm strap incision is made 1 cm medial to the AC joint. The acromial end of the coracoacromial ligament is detached, and the ligament is dissected free to the coracoid process. The lateral one centimetre of the clavicle is removed in an oblique fashion so that the inferior part of the oblique osteotomy overlies the coracoid process. The clavicle is held in an anatomic position relative to the coracoid and traction is applied to the coracoacromial ligament. The proper length is selected to maintain the reduction. Number 1 nonabsorbable nylon is placed in the ligament. Two small drill holes are made in the superior cortex of the clavicle, the suture material is passed through them, and the coracoacromial ligament is pulled into the medullary canal of the clavicle, securing the reduction. The repair is reinforced with three double strands of number 2 PDS sutures passed around the clavicle and underneath the coracoid and knotted anteriorly.

**Figure 3:**
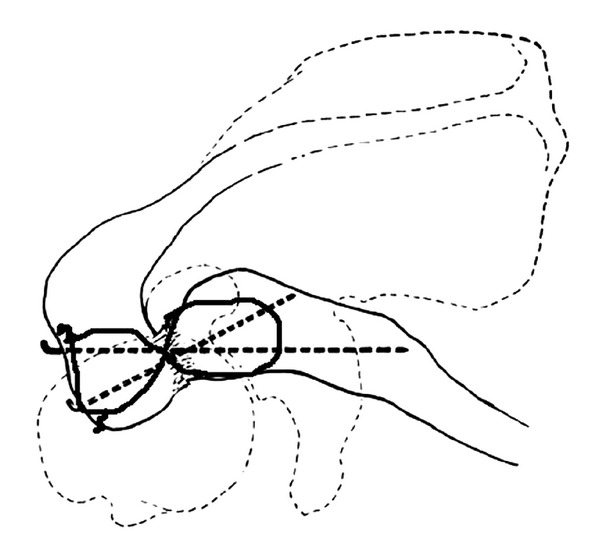
ORIF with TBW procedure.

 ORIF with TBW (Figure 3) and repair of CC ligament was used in the second group. All the patients had Type 3 ACJ dislocations and had relatively high physical demand in terms of their occupational/recreational activities. All the patients were given both operative and nonoperative options and all of them preferred operative treatment. The main exclusion criterion was delayed presentation of more than 6 weeks after the injury. Anterior curved approach to expose the ACJ, the lateral end of the clavicle, and the coracoid process was performed. The CC ligament status was defined. 4 patients had midsubstance tear of the CC ligament and 3 patients had avulsion of CC ligament from clavicle. Heavy absorbable sutures for CC ligament repair was passed before the AC Joint was reduced. 5 patients including the 3 with CC ligament avulsions required bone anchor sutures for a robust repair. Once the AC Joint was reduced, TBW with two 2 mm criss-cross K wires and 18 gauge (1.2 mm) steel wire in a figure of eight configuration was done. The CC ligament repair was then completed by tying the sutures.

## 4. Results

 18 cases of Rockwood Type 3 ACJ Dislocation had surgical stabilization over a period of 10 years. In the first group (delayed ligament reconstruction for failed non operative management with modified WD procedure) we had 11 cases (Table 1). In the second group (early ligament repair and ORIF with TBW) we had 7 cases (Table 2). TBW procedures were done for the acute injuries (mean interval between injury and surgery—10 days) and modified WD procedures were done for the chronic injuries (mean interval between injury and surgery—26 months) (Table 3). Mean age of the entire group was 31 years (16 to 59). 70% were males. We had 3 students. The rest were employed in various types of jobs ranging from office work to heavy duty manual work. Mechanism of injury was fall in 60%. Dominant shoulder was injured in 11 cases and nondominant in 7. Reasons for operative management in group 1 (modified WD) were pain and weakness of shoulder.

### 4.1. Complications

Only 1 patient out of 11 (9%) in the WD group had any postoperative complication. This 48-year-old gardener had shoulder pain 8 months after WD procedure but had good oxford shoulder score (58) at five and a half years post operatively. 5 out of 7 (71%) patients in the TBW group had post-operative complications. All 5 patients who developed post operative complications had their metal work removed.

### 4.2. Oxford Shoulder Score

Irrespective of the type of procedure/post operative complications all the 18 patients had good Oxford shoulder score, ranging from 50–60.

### 4.3. Patient Satisfaction

All patients, irrespective of the procedure, felt that they had full strength on their involved shoulder compared to the normal side. All patients except one were satisfied with the appearance of the shoulder. The WD patient who was not happy with the cosmetic result was recorded to have a repeat injury when he fell out of the bed 6 weeks postsurgery. One TBW patient who required repeat procedure had snapped wires 4 weeks post operative due to lack of compliance with post op regime. All patients except the pro ice hockey player who had TBW were able to return to their preinjury level of activity.

## 5. Statistical Analysis

We used SPSS software v 17.0. *P* value ≤0.05 was considered as statistical significance. Using unpaired *t*-test the TBW group and modified WD group were found to be comparable in terms of age and duration of clinical followup and telephone review. Mean age difference between the two groups: 1.6 yrs (*P* value 0.77). Mean difference in the duration of clinical followup is 1.87 months (*P* value 0.528). Mean difference in the duration of telephone follow up is 0.4 years (*P* value 0.079). on Comparing the long-term functional outcome based on Oxford shoulder score in the two groups by unpaired *t*-test has given a *P* value of 0.0504. Hence statistically no significant difference in long-term functional outcome was noted between the two groups.

## 6. Discussion

Over the past 30 years, many authors have supported nonoperative management for complete ACJ dislocations [7, 12–14]. Patients are treated conservatively even in cases of severe displacement [5, 7]. Systematic review by Spencer has concluded that non-operative treatment is superior to traditional operative treatment in the management of Grade III ACJ dislocation [15]. This conclusion was based on low level evidence that shows no better outcome among those treated surgically when compared to nonoperative treatment. Operative management was also associated with higher complication rates, longer convalescence, and longer time away from work and sport. Meta-analysis by Phillips has concluded that operative management is not recommended for Rockwood et al. Type III injury [3].

 However 20% to 40% of patients treated conservatively after an acute AC joint dislocation have unsatisfactory results, with residual pain during shoulder motion, paresthesia, loss of strength and fatigue with overhead activities, and/or cosmetic concerns [16, 17]. To highlight the controversy further, early ACJ fixations were supported by the following studies. 15 cases of ORIF reviewed by Roper and Levack has 100% good results [6]. Prospective RCT of Phemister procedure in 39 patients by Larsen et al. has 97% good results [5]. Comparison of various methods of internal fixation of AC joint has shown that K wire with tension band wiring gave the best results but required a more extensive operation for removal of implants [18]. 11 out of 14 patients with symptomatic complete ACJ dislocation treated by CC and AC ligament reconstruction with TBW had excellent to good results [19]. 

 Gohring et al. has shown higher risk of complications for TBW with K wires (43%) in treating complete ACJ dislocations [20]. Our study has confirmed the same. We had 71% of early postoperative complications in TBW group. But the long-term functional results of ORIF with TBW (early repair) were comparable with that of Modified WD Reconstruction for chronic ACJ dislocations.

Success rate of WD procedure ranged from 78 to 95% in various studies. 29 cases of WD procedure reviewed by Warren-Smith and Ward has 95% good result [21]. 9 cases of modified WD procedure by Copeland and Kessel has 89% good result [11]. 11 cases of Dacron coracoclavicular loop fixation by Bargren et al. has 91% good result [16]. In their original series Weaver and Dunn reported a failure rate of 28%, and poor results have been reported in other series, with loss of reduction after surgery because of stretch or pullout of the transferred Coracoacromial ligaments [5, 22].

A recent survey of over 500 members of the American Orthopaedic Society for Sports Medicine indicated that more than 80% of respondents prefer nonoperative treatment as initial management and delayed reconstruction in the event of failed conservative management was recommended [13]. Systematic review by Trainer et al. has concluded that patients with a grade III AC separation qualify for surgical reconstruction after a failed 3-month course of non-operative management as defined by persistent symptoms [23]. Our study also supports this conclusion due to the fact that modified WD reconstruction has less postoperative complications and hence better short-term functional outcome. WD reconstruction being a soft tissue procedure using biodegradable materials never required a second surgery.

## 7. Conclusion

While the debate on effectiveness of operative management for Type 3 ACJ dislocation continues, we would like to conclude that ORIF with TBW has high risk of post operative implant-related complications. These complications could be avoided by using a different type of fixation/implant (e.g., hook plate). Both early repair with internal fixation and delayed reconstruction with modified Weaver-Dunn procedure give comparable long-term functional outcome in Type 3 ACJ dislocation.

We recommend modified WD procedure for failed conservative management of Grade 3 ACJ dislocation for the following reasons.

(1) Better short-term functional outcome and hence faster recovery.

(2) No need for a second surgery and hence they impose less financial burden for the hospital as well as the patient.

## Figures and Tables

**Table 1:** Group 1 (modified WD procedure).

Age	Sex	Occupation	Mechanism of injury	Injured side	Interval b/w injury and surgery (months)	Complications	Total duration of followup (years)	Oxford shoulder score	Strength	Appearance	Return to preinjury level of activity
28	M	Engineer	Rugby	Dominant	18	Nil	6.5	57	Full	Satisfactory	Yes
35	M	Building site manager	RTA	Dominant	2	Rolled out of bed 6 weeks postop.? rupture	6	50	Full	Not satisfactory	Yes
39	F	Home care assistant	Fall	Dominant	3	Nil	5	60	Full	Satisfactory	Yes
35	M	Mechanic	Motor Cross Racing	Dominant	5	Nil	6.3	60	Full	Satisfactory	Yes
59	F	House wife	Fall	Non-dominant	5	Nil	6	56	Full	Satisfactory	Yes
43	M	Landscape gardener	Fall	Non-dominant	16	Shoulder pain 8 months postop.	5.6	58	Full	Satisfactory	Yes
22	M	Police officer	Rugby	Non-dominant	18	Nil	6.6	58	Yes	Satisfactory	Yes
16	F	Student	Fall	Non-dominant	24	Nil	6.6	58	Full	Satisfactory	Yes
18	M	Student	Fall	Dominant	24	Nil	6.6	58	Full	Satisfactory	Yes
31	M	Welder	Fall	Dominant	48	Nil	6.6	58	Full	Satisfactory	Yes
23	M	Student	Fall	Dominant	120	Nil	6.6	58	Full	Satisfactory	Yes

**Table 2:** Group 2 (ORIF with TBW procedure).

Age	Sex	Occupation	Mechanism of injury	Injured side	Interval b/w injury and surgery (days)	Complications	Metal work removal	Total duration of followup (years)	Oxford shoulder score	Strength	Appearance	Return to preinjury level of activity
26	M	Tennis coach	Skiing Accident	Dominant	1	Nil	3 months	6	56	Full	Satisfactory	Yes
38	F	House Wife	Fall	Dominant	2	Nil	9 months	7	54	Full	Satisfactory	Yes
18	M	Radio rental assistant	Fall	Non-dominant	5	Wire snapped 3 weeks postop. (patient non-compliant) Redo TWB at 4 weeks postop. 2 year postop. Decreased Range of Movement	No	7	54	Full	Satisfactory	Yes
26	F	Horse groomer	Fall	Dominant	12	Nil	2 months	6	56	Full	Satisfactory	Yes
44	M	Gym instructor	Football	Dominant	30	Impingement	No	7	54	Full	Satisfactory	Yes
33	M	Engineer	Fall	Non-dominant	35	2 months postop. Broken K wires (rolled over in bed) and shoulder pain	3 months	6	55	Full	Satisfactory	Yes
26	M	Sole trader	Rugby	Non-dominant	40	Migration of K wire, Removed 2 months postop.	2 months	6	54	Full	Satisfactory	No

**Table 3:** Comparison of the two groups.

	Modified WD	ORIF with TBW
Number	11	7
Mean age (range)	31.7 (18 to 44)	30.1 (16 to 59)
Sex (male : female)	8 : 3	5 : 2
Mean Interval between injury and surgery	26 months	10 days
Complications	1/11 (9%)	5/7 (71%)
Mean duration of clinic followup	5.4 months	7.3 months
Mean duration of total followup (telephone review)	6 years	6.4 years
Mean Oxford shoulder score at telephone review	57.1	54.7
Patient satisfaction		
Strength	100%	100%
Appearance	91% (10/11)	100%
Return to preinjury level of activity	100%	86%
